# Effect of oxygenation and temperature on glucose-xylose fermentation in *Kluyveromyces marxianus* CBS712 strain

**DOI:** 10.1186/1475-2859-13-51

**Published:** 2014-04-08

**Authors:** Lorenzo Signori, Simone Passolunghi, Laura Ruohonen, Danilo Porro, Paola Branduardi

**Affiliations:** 1 University of Milano Bicocca, Piazza della Scienza 2, 20126 Milan, Italy; 2 VTT Technical Research Centre of Finland, Espoo FI-02044 VTT, Finland; 3 Current address: Sacco srl, Via Manzoni, 29/A, 22071 Cadorago Co, Italy

**Keywords:** *Kluyveromyces marxianus*, Glucose fermentation, Xylose fermentation, Ethanol production, Oxygen requirement, Xylose reductase, Xylitol dehydrogenase

## Abstract

**Background:**

The yeast *Kluyveromyces marxianus* features specific traits that render it attractive for industrial applications. These include production of ethanol which, together with thermotolerance and the ability to grow with a high specific growth rate on a wide range of substrates, could make it an alternative to *Saccharomyces cerevisiae* as an ethanol producer. However, its ability to co-ferment C5 and C6 sugars under oxygen-limited conditions is far from being fully characterized.

**Results:**

In the present study, *K. marxianus* CBS712 strain was cultivated in defined medium with glucose and xylose as carbon source. Ethanol fermentation and sugar consumption of CBS712 were investigated under different oxygen supplies (1.75%, 11.00% and 20.95% of O_2_) and different temperatures (30°C and 41°C). By decreasing oxygen supply, independently from the temperature, both biomass production as well as sugar utilization rate were progressively reduced. In all the tested conditions xylose consumption followed glucose exhaustion. Therefore, xylose metabolism was mainly affected by oxygen depletion. Loss in cell viability cannot explain the decrease in sugar consumption rates, as demonstrated by single cell analyses, while cofactor imbalance is commonly considered as the main cause of impairment of the xylose reductase (*Km*XR) - xylitol dehydrogenase (*Km*XDH) pathway. Remarkably, when these enzyme activities were assayed *in vitro*, a significant decrease was observed together with oxygen depletion, not ascribed to reduced transcription of the corresponding genes.

**Conclusions:**

In the present study both oxygen supply and temperature were shown to be key parameters affecting the fermentation capability of sugars in the *K. marxianus* CBS712 strain. In particular, a direct correlation was observed between the decreased efficiency to consume xylose with the reduced specific activity of the two main enzymes (*Km*XR and *Km*XDH) involved in its catabolism. These data suggest that, in addition to the impairment of the oxidoreductive pathway being determined by the cofactor imbalance, post-transcriptional and/or post-translational regulation of the pathway enzymes contributes to the efficiency of xylose catabolism in micro-aerobic conditions. Overall, the presented work provides novel information on the fermentation capability of the CBS712 strain that is currently considered as the reference strain of the genus *K. marxianus*.

## Background

Modern yeast biotechnology places great emphasis on developing new traits in already established yeast cell factories as well as in identifying yeast species with novel traits or properties [1]. *Saccharomyces cerevisiae* has been used in biotechnological processes for centuries and it is therefore the best known and established yeast workhorse. However, in the last years, modern genetic and molecular techniques are promoting and facilitating the so-called non-conventional yeasts being reconsidered as alternative cell factories (as discussed in [2,3]). Among the non-*Saccharomyces* or non-conventional yeasts with potential for industrial applications are those belonging to the genus *Kluyveromyces.*

Within this genus *Kluyveromyces marxianus, Kluyveromyces lactis, Kluyveromyces aestuarii, Kluyveromyces dobzhanskii* and *Kluyveromyces wickerhamii* are highly related and appear clearly separated from the other *Kluyveromyces* species [4].

*K. lactis* is a model Crabtree-negative yeast that has been extensively investigated [5-7]. Since 1950s it has been used as a natural source of enzymes such as lactase/β-galactosidase, [8] and as a protein supplement in food [7]. From 1980s onwards, its easiness to genetic manipulations was recognized, and subsequently, suitable genetic tools have been developed, rendering it an efficient host for recombinant production [7,9,10].

*K. marxianus* has up to now received less attention from the scientific community [11], in spite of some very interesting characteristics such as the highest specific growth rate among eukaryotic microbes [12], the ability to grow at temperature up to 45-52°C [13-16], and the capacity of metabolizing a wide range of substrates including glucose, mannose, galactose, lactose, but also the pentose sugars xylose and arabinose [17]. These features could make *K. marxianus* an alternative to *S. cerevisiae* as an ethanol producer from lignocellulosic sugars [17-20]. Currently, *S. cerevisiae* plays the major role in ethanol production due to its high ethanol productivity, tolerance and its efficient hexose fermentation [21,22]. However, its inability to ferment xylose and other C5 sugars constitutes a major obstacle to efficient conversion of lignocellulose to ethanol. Moreover, thermotolerant yeast applicable for high temperature fermentation are expected to have potential in reducing cooling costs, increasing saccharification and fermentation rates, facilitating continuous ethanol removal and minimizing contaminations [13,16,23]. Also in this respect *S. cerevisiae* displays limitations, due to its very low fermentation efficiency at high temperature (>35°C, [24]). Therefore, the natural ability of *K. marxianus* to metabolize xylose, which is the main C5 sugar present in lignocellulosic hydrolysates and the second most abundant fermentable material [25], and its remarkable thermotolerance are particularly relevant when lignocellulose is used as raw material.

Strains belonging to the *K. marxianus* species have been isolated from a great variety of habitats, resulting in a genetic polymorphism which has been the focus of several studies [26,27].

This great variety, together with lack of published research on physiology, metabolism and biochemistry are possible reasons as to why a *K. marxianus* industrial strain, which could constitute a real alternative to *S. cerevisiae* for ethanol production has not been developed yet.

The strain CBS712 is currently considered as the reference strain of the genus *K. marxianus*[3] being the most studied also at the genomic level, with about 20% of its genome randomly sequenced [28]. Despite its ethanol production yield being very low compared to that of other strains of the same species, the fact that its genome is partially available, offers the possibility of future *in silico* analyses based on additional wet lab data on its metabolic capabilities.

In the present study batch fermentations under different temperatures and oxygen supplies with *K. marxianus* CBS712 were performed: the potential for xylose utilization and ethanol production was investigated, together with quantitative measurements of biomass formation, substrate consumption and external metabolite accumulation. Cell viability and oxidative stress response to the process conditions were additionally monitored by flow cytometric analyses. It has been reported that *K. marxianus* CBS712 can assimilate xylose but its ability to produce ethanol from xylose is coupled to oxygen feed [25]. Interestingly, the *in vitro* activity measurements of xylose reductase (*Km*XR) and xylitol dehydrogenase (*Km*XDH) demonstrated that the more the oxygen supply was restricted, the more the respective activities diminished, but with no corresponding decrease of mRNA levels. Therefore, the cofactor imbalance of the *Km*XR/*Km*XDH pathway, particularly pronounced under oxygen limitation [29], seems not the only explanation for the limited capacity of xylose fermentation in the *K. marxianus* CBS712 strain.

## Results

### Growth and fermentation profiles of *K. marxianus* CBS712 at 30°C with different inlet oxygen concentrations on mixture of glucose and xylose

The *K. marxianus* strain CBS712 was cultivated in batch mode in minimal defined medium [30] with xylose (20 g L^−1^) and glucose (20 g L^−1^) as carbon and energy source to evaluate the eventual sugar co-consumption, highly desirable for lignocellulosic second-generation ethanol production. Fermentations were performed at 30°C, pH 5 and followed for 70 hours. The inlet gas flow rate was maintained constant at 0.2 vvm (corresponding to 0.3 L min^−1^). The effect of three different concentrations of oxygen in the inlet gas flow, 20.95%, 11.00% and 1.75%, were monitored. For all the conditions studied, the dissolved oxygen fraction decreased to zero within 10 hours of growth. It has been reported that some strains of *K. marxianus* do not grow in anoxic conditions (as reviewed by [26]), while others do [31]. For this reason, at first it was verified that the model strain selected for the present study did not grow under anaerobic batch conditions (data not shown): therefore, fully anaerobic cultures were not assessed.

**Figure 1:**
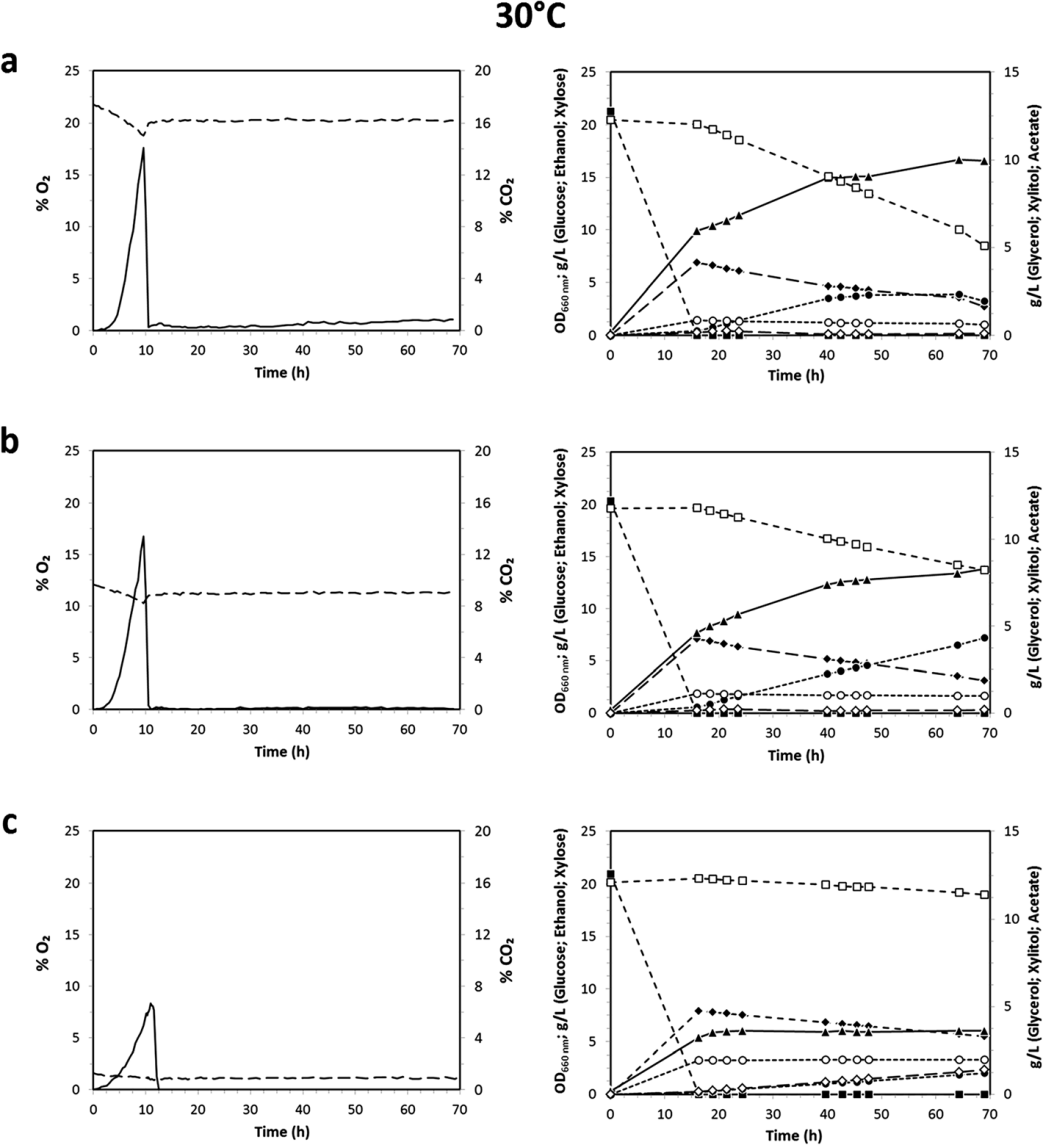
**Fermentation profiles of *K. marxianus *CBS712 at 30°C.** Fermentation profiles of *K. marxianus* CBS712 grown at 30°C under different concentrations of inlet oxygen: 20.95% **(a)**, 11.00% **(b)**, and 1.75% **(c)**. Left panels: O_2_ (%; dashed line) and CO_2_ (%; continuous line) profiles. Right panels: Biomass (OD_660_; ▲), Glucose (g L^−1^; ■), Ethanol (g L^−1^; ♦), Xylose (g L^−1^; ☐), Acetate (g L^−1^; **●**), Glycerol (g L^−1^; ○) and Xylitol (g L^−1^; ◊). Results are average values of three replicates where the deviation from the mean value was always less than 5%.

Figure 1 (left panels) shows the gas profiles of the three processes. The profiles of both O_2_ and CO_2_ over time were clearly related to the oxygen concentration of the inlet gas flow. Those of CO_2_ deserve some attention. Indeed, not only the total amount of CO_2_ produced, but also the maximum peak of production clearly appeared correlated with the amount of oxygen supplied in the gas flow. With the highest oxygen supply (Figure 1, left panel a), the peak of CO_2_ production was reached after 9.5 ± 0.2 hours from the inoculum, but only after 11.3 ± 0.2 hours with the lowest oxygen supply (left panel c). These differences were reflected on the profiles of sugar consumption and product accumulation as deduced by the measured samples, Figure 1, right panels. First of all, the production of biomass (closed triangles) was directly related to the oxygen supply, being proportionally lower for the lower concentrations of the inlet oxygen. Furthermore, and as already reported in literature, the CBS712 strain exhibited a delayed utilization of xylose (open squares), which was never significantly consumed until complete glucose (closed squares) exhaustion. Overall, also the xylose consumption appeared related to the concentration of the inlet oxygen, decreasing with the decrease of the oxygen supply: indeed, the amount of xylose consumed after 70 h of fermentation was about 12 g L^−1^ and 5.9 g L^−1^ with 20.95% and 11.00% of oxygen, respectively, and only about 1.5 g L^−1^ with 1.75% of oxygen (right panels a, b, c). The peaks of CO_2_ production (left panels) seemed to correlate with the onset of xylose consumption (or more precisely, with the glucose consumption being completed, see also below). Therefore, both biomass accumulation and the rate of sugar utilization were progressively decreased as the stringency of the oxygen supply increased (Table 1).

**Table 1:** Sugars consumption rates and ethanol production rates

**T**^**a**^**(°C)**	**O**_**2**_^**b**^**(%)**	**Volumetric consumption and production rates (gL**^**−1**^ **h**^**−1**^**)**							**Yields (gg**^**−1**^**)**			
		**Glucose**	**Xylose**	**Ethanol**	**Biomass**	**Glycerol**	**Acetate**	**Xylitol**	**Biomass**	**Glycerol**	**Acetate**	**Xylitol**
30°C	20.95	2,23	0,21	0,77	0,08	n.d.^c^	0,04	n.d.^c^	0,17	0,02	0,07	n.d.^c^
	11.00	2,13	0,11	0,79	0,07	0,01	0,07	n.d.^c^	0,17	0,03	0,16	n.d.^c^
	1.75	1,94	0,03	0,76	0,03	0,02	0,02	0,02	0,09	0,08	0,05	0,79
41°C	20.95	3,22	0,14	0,15	0,05	0,01	0,13	n.d.^c^	0,10	0,03	0,26	n.d.^c^
	11.00	3,16	0,15	1,18	0,05	0,01	0,11	n.d.^c^	0,11	0,04	0,24	n.d.^c^
	1.75	2,80	0,05	1,12	0,03	0,02	0,03	0,04	0,08	0,09	0,08	0,75

^a^T, temperature.

^b^O_2_, inlet oxygen.

^c^n.d., not detectable.

Sugar (glucose and xylose) consumption rates, production rates (biomass, ethanol, glycerol, acetate and xylitol) and yields (biomass, glycerol, acetate and xylitol) at different temperature and oxygen supply. Glucose consumption rates were calculated during the CO_2_ production phase, while xylose consumption rates were calculated starting from the first point after glucose exhaustion until the last point of the fermentation. Ethanol production rates relate only to the glucose consumption phase since on xylose consumption phase, in all the tested conditions, no significant ethanol production was observed. Yields and volumetric production rates of biomass, glycerol and acetate were calculated based on the total consumed sugars, while xylitol yields and volumetric production rates were calculated based on the consumed xylose only.

The results shown are average values of three replicates where the deviation from the mean value was always less than 3%.

Ethanol production (closed diamonds) was in essence only observed during the exponential growth on glucose. Interestingly, similar amounts of ethanol were measured under the three different conditions. We can safely speculate that the highest level of ethanol accumulation correlated with the peak of CO_2_ production (compare left with right panels).

As shown in Figure 1 (right panels), no noticeable ethanol production was detected during the xylose consumption phase of the batch fermentations. However, the CBS712 strain was able to produce ethanol from xylose, even if with very low yields, between 0.08 and 0.09 g g^−1^. These experiments were performed in shake flasks with xylose (20 g L^−1^) as the sole carbon source, both at 30°C and 41°C, with similar results (data not shown).

With the exhaustion of glucose and the onset of xylose consumption a decrease in the ethanol concentration was observed, being the trend of xylose and ethanol profiles very likely an indication of their co-consumption. In fact, evaporation tests (data not shown), performed under the same operative conditions demonstrated that stripping was not sufficient to explain the decrease in the ethanol concentration. This suggests that the CBS712 strain grows on ethanol after glucose exhaustion: in detail, it consumed ~1 g L^−1^ and ~3 g L^−1^ of ethanol with the lowest (1.75% O_2_) and the highest (20.95%) oxygen supply, respectively.

The lower the oxygen supply, the higher was the glycerol (open circles) and xylitol (open diamonds) accumulation. Production of glycerol can be described as an alternative way to ethanol of recycling NADH to NAD^+^[32]. However, as already reported in literature for oxygen-limited conditions [29,33], the inefficient regeneration of NAD^+^ led also to xylitol accumulation. Acetate (closed circles) was produced in consistent amounts with high and medium oxygen supplies (Figure 1, right panels a and b), while its accumulation was limited under 1.75% oxygen inlet (panel c).

### Growth and fermentation profiles of *K. marxianus* CBS712 at 41°C with different inlet oxygen concentrations on mixture of glucose and xylose

**Figure 2:**
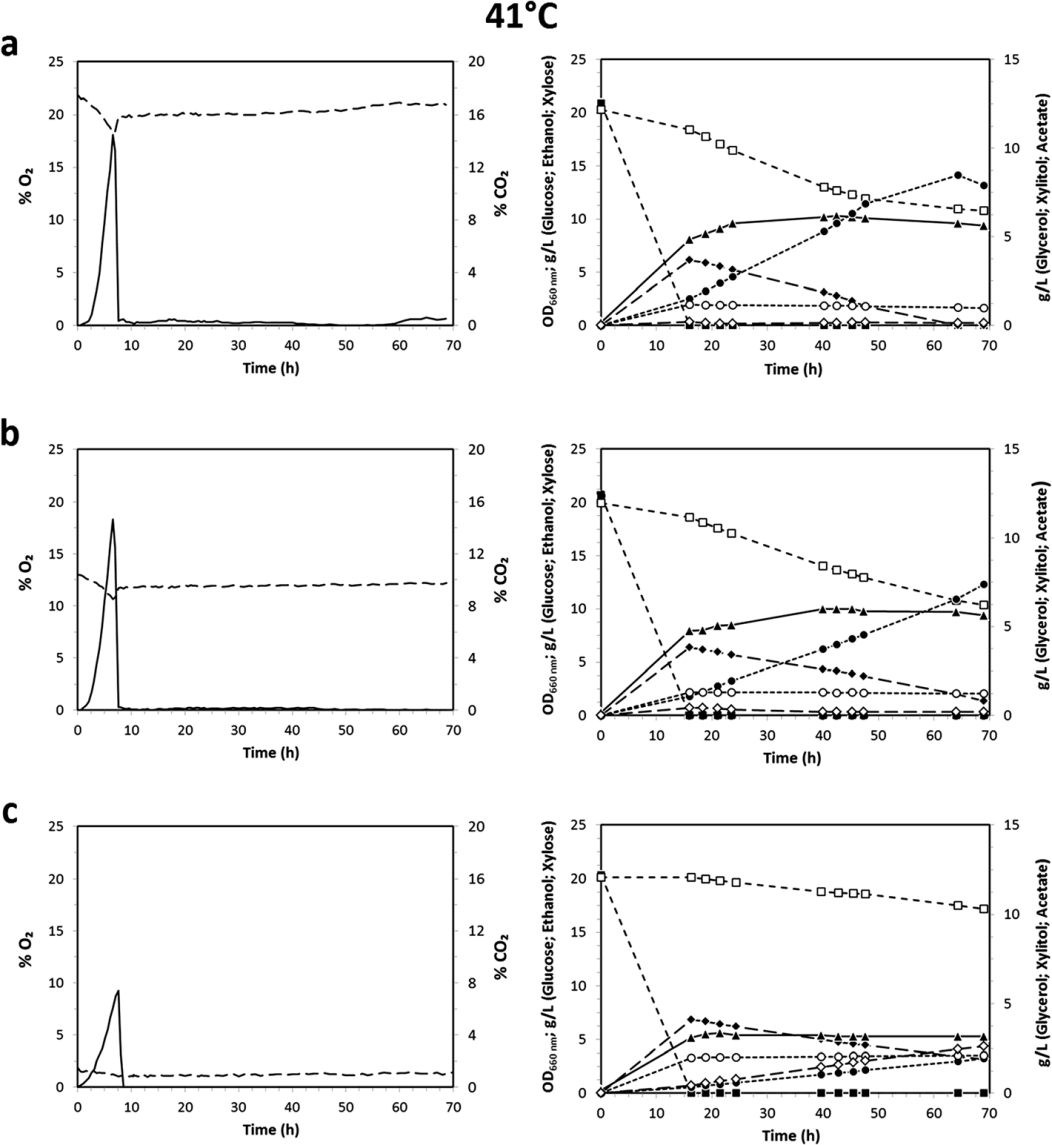
**Fermentation profiles of *K. marxianus *CBS712 at 41°C.** Fermentation profiles of *K. marxianus* CBS712 grown at 41°C under different concentrations of inlet oxygen: 20.95% **(a)**, 11.00% **(b)**, and 1.75% **(c)**. Left panels: O_2_ (%; dashed line) and CO_2_ (%; continuous line) profiles. Right panels: Biomass (OD_660_; ▲), Glucose (g L^−1^; ■), Ethanol (g L^−1^; ♦), Xylose (g L^−1^; ☐), Acetate (g L^−1^; **●**), Glycerol (g L^−1^; ○) and Xylitol (g L^−1^; ◊). Results are average values of three replicates where the deviation from the mean value was always less than 5%.

To evaluate the influence of temperature, the experiments described were repeated at 41°C, Figure 2. From a general point of view, the gas profiles showed similar trends (compare Figure 1 and Figure 2, left panels). However, at 41°C the CO₂ production rates (and therefore the glucose consumption rates) were markedly faster than at 30°C. Consequently, the onset and efficiency of xylose consumption was also more rapid at oxygen supply of 20.95% and 11.00% than at the lower temperature assessed. Once more, the kinetic profile suggests an apparent glucose-xylose co-consumption, very likely only a consequence of the sampling time, as also verified in shake flasks experiments (data not shown). Remarkably, at 41°C the biomass production (closed triangles) with high or medium oxygen supply was significantly lower after glucose exhaustion during the phase of xylose consumption.

As shown in Table 1, and as previously suggested by the CO₂ profiles (Figures 1 and 2, left panels), both the amount of oxygen supplied (1.75%, 11.00% and 20.95% of O_2_) and temperature (30 and 41°C) affected the overall glucose consumption rate. In particular, at 41°C and independently from the oxygen supply, the glucose consumption rates were faster than at 30°C: at 41°C the lowest glucose consumption rate was ~2.80 g L^−1^ h^−1^ (1.75% of O_2_) while at 30°C the highest consumption rate was ~2.23 g L^−1^ h^−1^ (20.95% of O_2_). Overall, the glucose consumption rates were more influenced by changes in temperature than by changes in the oxygen supply.

Also at the higher temperature ethanol was produced only during the exponential growth on glucose and at similar levels. However, at 41°C the ethanol production rates were markedly faster than at 30°C, as expected by the observed glucose consumption rates (Table 1).

Interestingly, the amount of oxygen supplied did not significantly affect the ethanol production rates, even if at both temperatures (30 and 41°C) the same trend was observed: the lower ethanol production rates were observed with 1.75% of inlet oxygen (~0.76 g L^−1^ h^−1^ and ~1.12 g L^−1^ h^−1^ at 30 and 41°C, respectively) while the highest production rates were observed with 11.00% of inlet oxygen (~0.79 g L^−1^ h^−1^ and ~1.18 g L^−1^ h^−1^ at 30 and 41°C, respectively). Also at 41°C, evaporation tests (data not shown) demonstrated that stripping, even if higher than that observed at 30°C, was not sufficient to explain the decrease in ethanol concentration(s), suggesting a co-consumption of ethanol and xylose.

Table 1 also reports the xylose consumption phase after glucose exhaustion: while at 30°C the xylose consumption rate was by far the highest with 20.95% of inlet oxygen (~0.21 g L^−1^ h^−1^), at 41°C significant differences were not observed between high or medium oxygen supply (~0.14 g L^−1^ h^−1^ and ~0.15 g L^−1^ h^−1^, respectively).

These values underline once more that both the oxygen feeding and temperature are key parameters for the ability of CBS712 strain to consume xylose.

Like previously observed at 30°C, xylitol (open diamonds) and glycerol (open circles) accumulation increased at the lowest inlet of oxygen supply. Overall, the highest concentration of xylitol was assessed at 41°C with 1.75% of inlet oxygen, with consumption of 3 g L^−1^ of xylose and accumulation of 2.7 g L^−1^ of xylitol, corresponding to a yield of 0.75 gg^−1^. In the same condition the highest glycerol accumulation and yield (2.1 g L^−1^ and 0.09 gg^−1^, respectively) was also measured (Table 1).

Finally, at 41°C greater accumulation of acetate compared to the fermentations at 30°C was observed (Figures 2 and 1, closed circles, respectively). As an example, with 20.95% of oxygen, the highest acetic acid production and yield were 8.5 g L^−1^ and 0.26 gg^−1^ compared to 2.3 g L^−1^ and 0.06 gg^−1^ at 41°C and 30°C, respectively (Table 1).

### Viability and intracellular ROS accumulation assessed by flow cytometric analyses

**Figure 3:**
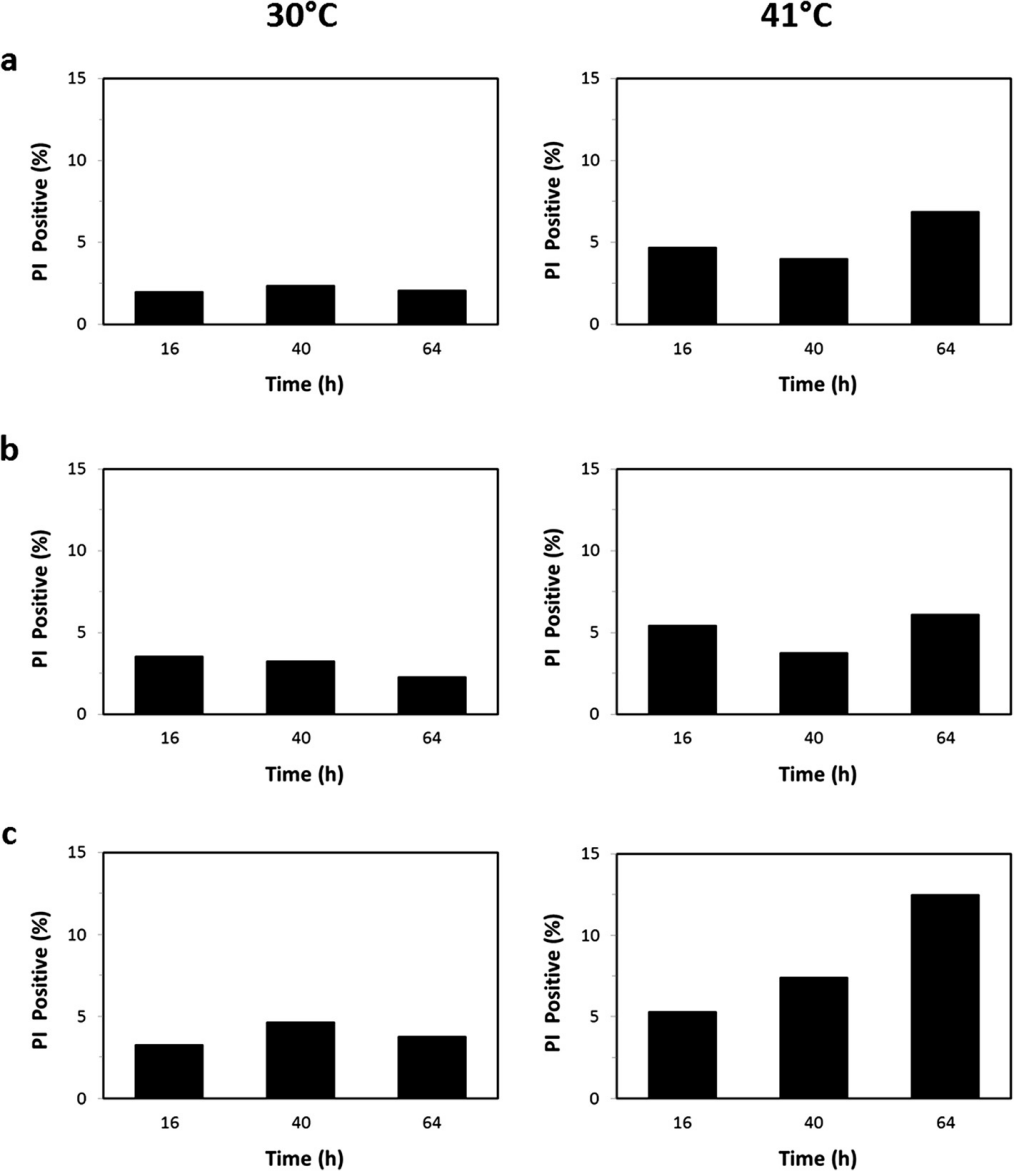
**Cell viability.** Percentage of damaged/dead cells during fermentations at 30°C (left panels) and 41°C (right panels) under different concentrations of inlet oxygen: 20.95% **(a)**, 11.00% **(b)** and 1.75% **(c)**. The results shown are average values of three replicates where the deviation from the mean value was always less than 5%.

Cellular viability, or more generally active metabolism and cellular integrity, can strongly influence substrate uptake and production rates of biomass and metabolites. Therefore, a set of analyses at single cell level was performed to evaluate this parameter during the fermentation processes previously described. Cells were collected at different time points (T = 16, T = 40 and T = 64 hours after inoculation, see Figures 1, 2), stained with propidium iodide (PI) and analysed by flow-cytometry to detect severely damaged/dead cells ([34], see Materials and methods). In Figure 3 the percentages of severely damaged/dead cells in the different conditions tested are reported.

Generally speaking, the percentages of PI positive cells were very low, independent from the fermentation parameters. In particular, at 30°C such fraction never exceeded 5% of the whole population. At 41°C the percentages were a bit higher, slightly rising along the time and with the decreasing oxygen supply. Overall, the percentages of damaged cells appeared negligible in respect to the whole population, suggesting that the imposed process conditions did not severely affect the cell viability/integrity. Consequently, low viability does not seem to be the reason for the low xylose consumption rates.

**Figure 4:**
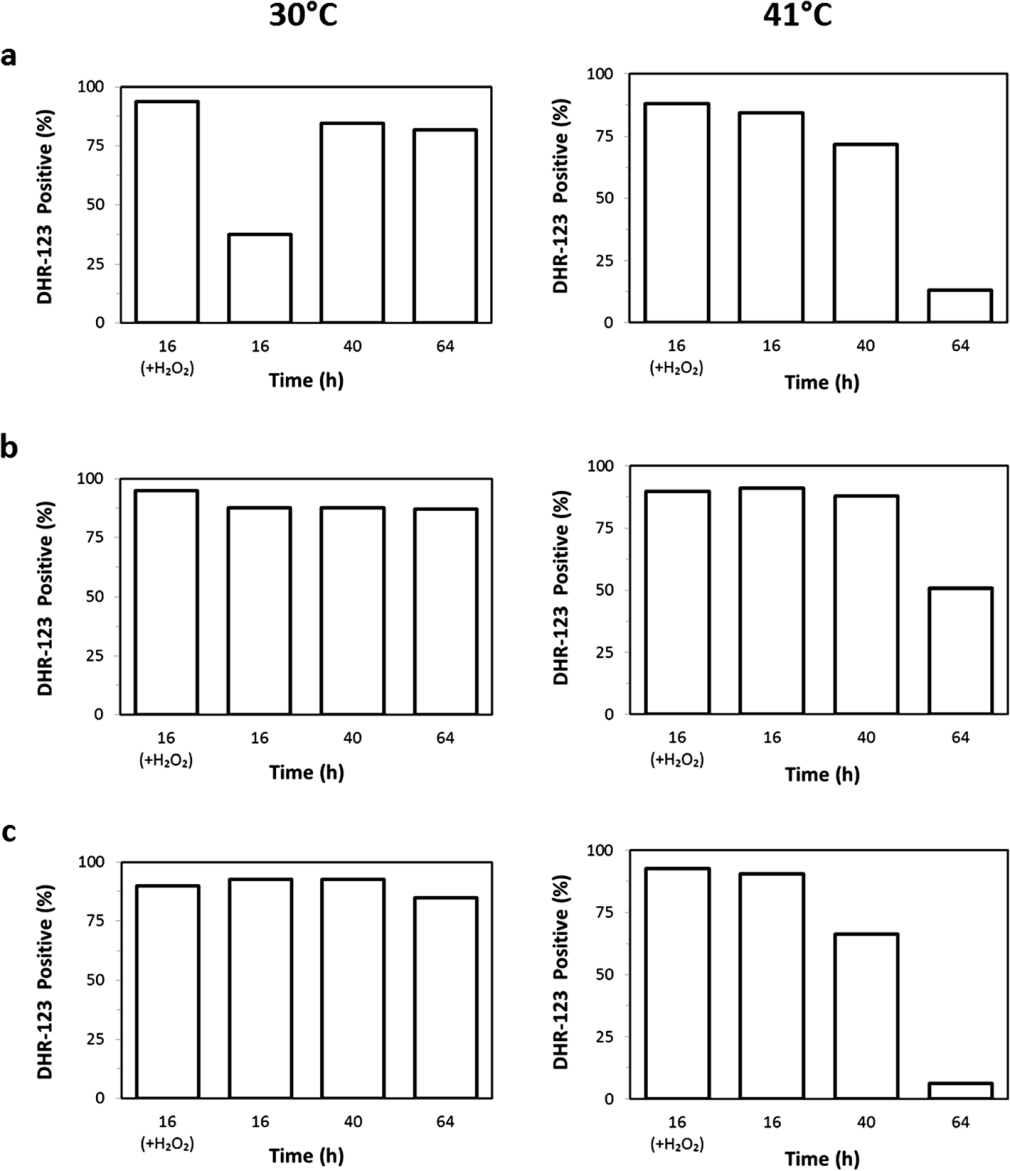
**Intracellular ROS accumulation.** Percentage of cells that accumulate ROS during fermentations at 30°C (left panels) and 41°C (right panels) under different concentrations of inlet oxygen: 20.95% **(a)**, 11.00% **(b)**, and 1.75% **(c)**. For each condition, an example of positive control (*i.e.*: cells treated with 6 mM hydrogen peroxide) is reported. The results shown are average values of three replicates where the deviation from the mean value was always less than 5%.

Cells were also stained with dihydrorhodamine-123 (DHR-123) to detect intracellular accumulation of reactive oxygen species (ROS; [35], see Materials and methods, Figure 4). Surprisingly, at 30°C a high percentage of cells accumulated ROS under all the process conditions tested, Figure 4 left panels (being lower but still significant only during the first time point measured with the highest oxygen inlet). In contrast, at 41°C a progressive decrease in the comparably high intracellular ROS accumulation was observed, right panels. These trends were also confirmed by fluorescence microscopy analyses (data not shown). Data currently available are not sufficient to unambiguously explain either the high ROS accumulation both at 30°C and 41°C, or the decrease over time in ROS accumulation observed at 41°C. However, these results are further evidence of the different cellular metabolism at 30°C and 41°C, as previously commented (Figure 1*vs.* Figure 2).

### Activity and transcript level of xylose reductase (*Km*XR) and the xylitol dehydrogenase (*Km*XDH)

Xylose reductase (*Km*XR) and xylitol dehydrogenase (*Km*XDH) are the two key enzymes of xylose metabolism in *K. marxianus*[25,29,36], like in most other xylose-consuming yeast.

Aiming at further understanding of the xylose consumption pathway in the *K. marxianus* CBS712 strain, the activity of these two enzymes was determined during the xylose consumption phase of the batch cultures studied, Table 2. To simplify the interpretation of the data, only the two extreme conditions (20.95% and 1.75% inlet oxygen) are reported, being the data obtained with 20.95% and 11.00% inlet oxygen very similar. At both temperatures and all time points, the specific *Km*XDH activities were always higher than the specific *Km*XR activities. Overall, it can be noticed that for both activities, independently from the temperature, the higher values related to the higher inlet oxygen (Table 2). In detail, *Km*XR activity dropped almost to half when the inlet oxygen decreased from 20.95% to 1.75% at 30°C, while the reduction was less evident, if even present, at 41°C (where actually an increasing trend in the activity was observed). The inlet oxygen decrease (from 20.95% to 1.75%) determined a decrease also for the *Km*XDH activity. However, differently to what previously observed for the *Km*XR activity, the reduction was very dramatic at 41°C, approaching one order of magnitude. At 30°C, the initial decrease to less than one third (see data at early time point) was followed by an increase along the time, despite values never reaching the ones measured from samples collected from cultures at 20.95% of inlet oxygen.

**Table 2:** **Xylose reductase ( *Km *** **XR) and xylitol dehydrogenase ( *Km *** **XDH) activities**

**Inlet oxygen (%)**	**Time (h)**	**Enzyme activity (U mg**^**−1**^**)**		**Enzyme activity (U mg**^**−1**^**)**	
		**XR**	**XDH**	**XR**	**XDH**
20.95	16	6,4	45,2	6,6	39,0
	23	7,4	43,9	6,1	37,9
	40	8,5	47,6	5,0	37,1
1.75	16	3,6	13,7	3,5	4,2
	23	2,3	22,3	4,6	6,3
	40	4,3	37,0	5,9	7,6

Xylose reductase (*Km*XR) and xylitol dehydrogenase (*Km*XDH) activities of *K. marxianus* CBS712 grown at 30°C and 41°C under different inlet oxygen concentrations: 20.95% (a) and 1.75% (b) are average values of two replicates where the deviation from the mean value was always less than 3%.

It has to be highlighted that the enzymatic activities were assayed *in vitro*: this means that cofactor imbalance due to the different cofactor preferences of the two enzymes, which is commonly recognized as the limiting factor of this metabolic pathway under limited oxygen conditions [37], cannot be the explanation for the reduced activities measured, and the correlation with the fermentation profiles is not trivial. In fact, by looking once more at the data of the cultures at 30°C, both of the activities, in particular that of *Km*XDH, were lower at the two earlier time points assayed, but had increased at the last measurement point (40 h), while the xylose metabolism remained unchanged (see again Figure 1, open squares). On the other hand, from these data it can be concluded that *Km*XDH was the most affected enzyme by the oxygen supply, justifying in this case the previously described trends of xylitol accumulation (Figures 1 and 2 open diamonds): the higher accumulation of xylitol corresponded to the lower *Km*XDH specific activity.

Differences in transcription profiles could explain the trend of the enzymatic activities observed. Thus, transcript levels were determined at different time points (T = 16, T = 40 and T = 64 h) during the fermentations described in Figures 1 and 2. However, the *Km*XR and *Km*XDH expression profiles showed no significant variation at any of the time points studied at the two different temperatures (data not shown). This suggests that, not excluding the impairment determined by cofactor imbalance, post-transcriptional and/or post-translational regulation of the *Km*XR-XDH xylose pathway plays a role in determining the efficiency of xylose metabolism in micro-aerobic conditions in the CBS712 strain of *K. marxianus*.

## Discussion

The aim of the present study was to characterize the fermentation profiles of the *K. marxianus* CBS712 strain during growth in defined medium with glucose and xylose as carbon source, and with different oxygen supply mimicking the micro-aerobiosis conditions occurring in industrial processes. Moreover, to exploit the peculiar thermotolerance of this yeast, different operative temperatures were compared (see Materials and methods). These conditions, despite still far from a real process of second generation ethanol production, provide useful wet data for future investigations and *in silico* analyses. *K. marxianus* CBS712 strain was chosen even if its ethanol production yield is low compared to that of other strains of the same species because it represents the reference strain of the species and, to the best of our information, is the only *K. marxianus* strain whose genome has been partially sequenced [28,38].

The data collected allowed describing the metabolic differences occurring by changing either the temperature or the inlet oxygen of the fermentation process. A number of these differences are more directly related to the ethanol production here reported or can be commented in the view of a lignocellulose-based process for ethanol production. They can be summarized as follows: *i)* independently from the temperature, the more the oxygen supplied was reduced, the more the metabolism was attenuated, and in particular that of xylose; *ii)* independently from the oxygen supply, *K. marxianus* CBS712 was able to grow both at 30 and 41°C, with a faster overall metabolism at 41°C, as confirmed by the CO_2_ production rates, which were faster compared to those of fermentations performed at 30°C. As a possible consequence of an energy demanding condition, the biomass accumulation at 41°C was always lower than the corresponding biomass values reached at 30°C; *iii)* especially at the higher temperature, there was a significant increase in acetic acid accumulation that might be related to NADPH demand. In fact, it is well reported in literature that not only the pentose phosphate pathway, but also the acetaldehyde dehydrogenase can significantly contribute to the overall NADPH pool [39,40]. Additional data would be necessary to fully elucidate this point: however it should be noted that NADPH is the *Km*XR cofactor [41]. Moreover, since reducing power is required for the neutralization of reactive oxygen species [42,43], NADPH formation through the acetate pathway might be required for counteracting ROS accumulation. This correlates with the overall ROS decrease observed at 41°C over time (Figure 4); *iv)* it is evident that, independently both from the oxygen supply and from the temperature values, the xylose fermentation always followed the glucose consumption (being slower with the lower inlet oxygen, see above). Considering that in the tested conditions the ethanol accumulation was evident only during the glucose phase, it can be concluded that no detectable ethanol production derived from xylose; *v)* in the tested conditions, cell viability did not seem to be an issue in *K. marxianus* CBS712, as demonstrated by single cell analysis. This point was addressed since previous reports on fermentation at high temperature evidenced a rapid decrease of cell viability [13]. However, it is important to remember that a very high percentage of cells showed positive in ROS detection; *vi)* remarkably, the overall ethanol production was very similar in all the tested conditions.

Considering the favourable physiological traits of *K. marxianus*, glucose-xylose co-fermentation appears as the most urgent to be addressed for lignocellulose-based production. In this respect, the biochemical data well supported how, also in *K. marxianus*, impairment in xylose metabolism under micro-aerobic condition can be ascribed to the difference in cofactor preference between *Km*XR and *Km*XDH [25,29,41]. Zhang and co-authors [44] overexpressed in *K. marxianus* a NADPH-preferring xylose reductase and engineered its cofactor preference to revert it to NADH. Data undoubtedly demonstrate the beneficial effect of a higher *Km*XR specific activity on xylose fermentation, while the advantage of cofactor exchange remains dubious. Not in contrast with that, here we demonstrated that additional regulation may occur at post-transcriptional level, since *in vitro* measured activities of both *Km*XR and *Km*XDH were reduced together with inlet oxygen reduction, despite the fact that the preferred cofactors were supplied within the reaction mix. Additionally, no coherent differences in transcription levels of the genes encoding these two enzymes were measured. An alternative way of solving the problem was more recently proposed by Wang and co-authors [45], by substituting the native xylose pathway with the fungal one based on xylose isomerase, providing promising results. It would be very interesting to see if even in this engineered strain glucose represses xylose fermentation, as demonstrated in the native strain for different ratios of these two sugars [46]. Finally, it is interesting to underline once more the high or very high percentage of cells positive in ROS staining: this might prelude to the higher mortality registered when *K. marxianus* cells were grown in the presence of different pre-treated lignocellulose preparations [47,48], which are known to contain pro-oxidative inhibitors, such as acetic acid. In this respect, as already demonstrated for *S. cerevisiae*[49,50], improving strain robustness can certainly be seen as an additional manipulation target to be conducted for developing *K. marxianus* as a real possible alternative yeast for second generation processes of bio-production.

## Conclusions

The results presented provide data indicating that sugars fermentation in the *K. marxianus* CBS712 strain is affected both by oxygen supply and temperature. Xylose fermentation, and more importantly glucose and xylose co-fermentation, under limited oxygen supply is of particular relevance for second generation bioethanol production. Therefore, fermentation profiles and biochemical data on the key metabolic pathway are desirable information for the development of an efficient cell factory. We showed a direct correlation between the decreased efficiency to consume xylose with the reduced specific activity of the two main enzymes (*Km*XR and *Km*XDH) involved in its catabolism. Therefore, it is possible to hypothesize that in the CBS712 strain the efficiency of xylose catabolism in micro-aerobic conditions is influenced not only by the cofactor imbalance, but also by post-transcriptional and/or post-translational regulation of the key enzymes of the pathway. Overall, the presented work provides novel information on the fermentation capability of the CBS712 strain that is currently considered as the reference strain of the genus *K. marxianus*.

## Materials and methods

### Yeast strain and inoculum preparation

The *K. marxianus* strain used in this study was CBS712 (http://www.cbs.knaw.nl, alias NBRC 10005). Strain maintenance: the strain was overnight shake flask cultured in YPX medium (1% wv^−1^ yeast extract, 2% wv^−1^ peptone, 2% wv^−1^ xylose). Sterile glycerol was added to a final concentration of 20% (vv^−1^) and cells were stored in cryotubes at −80°C. Pre-cultures were always prepared transferring some cells from these stock tubes to YPX agar plates and inoculating single colonies (visible after 24 h of incubation at 30°C), in defined mineral medium [30] with glucose (20 g L^−1^) and xylose (50 g L^−1^) as carbon sources. One colony was transferred into a 100 mL shake-flask with 25 mL medium and incubated 16 h at 30°C with an agitation speed of 160 rpm. All 25 mL were transferred into a 250 mL shake-flask with 50 mL defined mineral medium with glucose (5 g L^−1^) and xylose (20 g L^−1^) as carbon sources, and incubated at 30°C with an agitation of 160 rpm until exponential growth phase was reached.

### Batch cultivations

Exponential phase shake-flasks cultures were used to inoculate 2 L bioreactors (Sartorius Stedim, BIOSTAT B), with an operative volume of 1.5 L, to a final absorbance of OD_660nm_ 0.3 (CDW about 0.1 g L^−1^) in defined mineral medium [30] with glucose (20 g L^−1^) and xylose (20 g L^−1^) as carbon and energy source. Stirrer speed was set at 250 rpm and the initial pH value at 5, maintained with 2 M NaOH. The inlet gas flow rate was 0.3 L min^−1^ (0.2 vvm), adjusted with two mass flow controllers (Bronkhornst®High Tech- EL-FLOW®Select), one for air and the other for nitrogen (N_2_). The two mass flow controllers were used to set the desired gas mixtures. Batch cultivations were carried out at 30 or 41°C and three different concentration of inlet oxygen were tested: 20.95%, 11.00% and 1.75% of oxygen. Overall, six different fermentation protocols were run in triplicate varying temperature (30 or 41°C) and inlet oxygen (20.95%, 11.00% and 1.75% of oxygen).

The composition of the fermenter off-gas was on-line measured by a gas analyzer (Omnitec). The gas analyser was always calibrated 24 h before starting a cultivation using synthetic air containing a defined concentration of CO_2_ (1%).

Samples (20 mL) were collected regularly from the bioreactor in vials; 1 mL was used for OD_660nm_ measurement_,_ after appropriate dilution; 1 mL was centrifuged at 4°C, 14.000 rpm, for 5 min and supernatants were collected and stored at −20°C for later determination of metabolites concentrations.

The remaining liquid cultures were divided as described later and harvested by centrifugation (2000 rpm × 10 min). Pellets were stored at −80°C for subsequent enzymatic and RTq-PCR analysis.

### Analytical methods

Cell growth was spectrophotometrically estimated as OD_660nm_ (Shimadzu UV-1800) and gravimetrically verified by measuring the cell dry weight (CDW g L^−1^). For the CBS712 strain we established that a CDW of 1 g L^−1^ correspond to an OD_660nm_ of 2.86. For dry weight determination, cells were filtered through a dried and pre-weighed 0.22 μm-pore membrane (Millipore, USA), washed twice with distilled water and dried in a microwave oven (180 W, 10 min). The sample volume filtered was 10 mL and the dry weight measurements were performed in duplicates for all samples.

Glucose, xylose, xylitol, glycerol, acetate, and ethanol concentrations were HPLC determined using a cation-exchange column (Bio-Rad Aminex® HPX-87H). The eluent was 5 mM H_2_SO_4_ pumped at 0.6 mL min^−1^ and column temperature was 45°C. Separated components were detected by a refractive-index detector and peaks were identified by comparing with known standards (Sigma-Aldrich, St Louis, MO, USA).

### Flow cytometric analyses

Dead or severely compromised cells were detected following Propidium Iodide (PI, Sigma-Aldrich CO., St. Louis, MO, USA) staining. Briefly, cell were washed twice with buffer (TrisHCl 50 mM, MgCl_2_ 15 mM, pH 7.7), resuspended in a PI solution (0.23 mM), incubated in the dark on ice for 20 min and then analysed by flow-cytometry.

Reactive oxygen species (ROS) were detected by Dihydrorhodamine-123 (DHR-123, Sigma-Aldrich CO., St. Louis, MO, USA) as previously described by [35]. Briefly, cells were incubated for 2 h with Dihydrorhodamine-123 (5 μg mL^−1^ from a 2.5-mg mL^−1^, stock solution in ethanol), washed twice with PBS buffer and then analysed by flow-cytometry.

For both PI and DHR-123 staining positive and negative controls were performed. PI positive control was settled by killing cells with ethanol; ROS positive control was settled by adding to the medium H_2_O_2_ 6 mM. Percentages shown in Figures 3 and 4 have been calculated by taking in account autofluorescence.

Samples were analysed using a Beckman Coulter FC-500 flow cytometer (Beckman Coulter, Fullerton, CA, USA) equipped with an Argon ion laser (excitation wavelength 488 nm, laser power 20 mW). The fluorescence emissions were measured through a 525–550 nm band pass filter (FL1 parameter) for DHR signals and through a 670 nm band pass filter (FL4 parameter) for PI signals. The sample flow rate during analysis did not exceeded 500 cells s^−1^. A total of 25.000 cells were measured for each sample. Data analysis was performed afterwards with *Cyflogic* software (PerttuTerho, Mika Korkeamäki, CyFlo Ltd).

### Protein extraction and enzyme assays

For total protein extraction, 10 mL of liquid culture was harvested by centrifugation (2000 rpm × 10 min) and washed twice with 10 mL of ice-chilled distilled water. Subsequently, cells were resuspended in 1 mL of distilled water, transferred in FastPrep® tube and harvested by centrifugation (10000 rpm × 5 min). Total soluble protein were obtained by three treatments (30 sec, speed 6) of beads beating (*Savant Bio 101 FastPrep*®), interspersed by 30 sec on ice, in Tris–HCl buffer (0.1 M and pH7.5). After extraction, the debris was pelleted by centrifugation (10000 rpm × 5 min at 4°C). Supernatants, containing soluble proteins, were transferred to clean tubes and stored at −20°C until required. Protein concentration was measured according to Bradford [51], with bovine serum albumin as the standard.

*Km*XR activity was spectrophotometrically measured by monitoring the oxidation of NAD(P)H at 340 nm [52] in a reaction mixture (1.0 mL) with the following composition: 0.1 M sodium phosphate buffer (pH7), 0.2 M xylose and 0.15 mM NAD(P)H. *Km*XDH activity was measured by monitoring the reduction of NAD^+^ at 340 nm [52] in a reaction mixture (1.0 mL) with the following composition: 0.1 M TrisHCl (pH7), 1 mM MgCl_2_, 50 mM xylitol and 5 mM NAD^+^. Reactions were started by the addition of substrate. The specific activities of the enzymes were expressed as U mg^−1^.

One unit of enzyme activity was defined as the amount of enzyme required to produce 1 μmol of NADH/NAD(P)^+^ per min under the assay conditions [53].

### Total RNA extraction, cDNA synthesis and RTq-PCR

For total RNA extraction, 5 mL of liquid culture was harvested by centrifugation (2000 rpm × 10 min) and washed twice with 5 mL of ice-chilled distilled water. Subsequently, cells were resuspended in 5 mL of distilled water and the required volume of cells was transferred into a 2 mL tube. Total RNA was extracted using the Aurum Total RNA Mini Kit (Bio-Rad, USA) according to manufacturer’s protocol. The concentration of total RNA was spectrophotometrically measured (Shimadzu UV-1800; OD_260nm_) using quartz cuvettes.

About 0.5 μg of total RNA was converted into cDNA by reverse transcription in a 20-μL reaction mixture using the iScriptcDNASyntesis Kit (Bio-Rad, USA) with the provided random primer mix (5X iScript reaction mix) following the recommended protocol.

The RTq-PCR was performed using the SsoAdvanced SYBR® Green Supermix (Bio-Rad, USA) according the manufacturer’s instructions. For XR and XDH primer design, sequences deposited as GU574744 and GU574813 were used, respectively. Primer-3 (http://primer3.ut.ee/) was used to design PCR oligonucleotides, listed in Table 3.

**Table 3:** Primers list

**Gene**	**Orientation**	**Oligonucleotide sequence**
XR	Fwd	5′-CAGAGCCCTTGAGAAGTTGG-3′
	Rev	5′-AAGGATAGTGGGCCGAAACT-3′
XDH	Fwd	5′-GCTGTAGAGCCAGGTGTTCC-3′
	Rev	5′-CAAATCCAGTACCGGCAAGT-3′
ATC1	Fwd	5′-AGCACCCAGTTTTGTTGACC-3′
	Rev	5′-AGGAGAAACCGGCGTAGATT-3′

Primers designed and used in the current study.

The PCR amplification conditions were as follows: initial denaturation for 30 sec at 95°C, PCR amplification for 40 cycles with denaturation for 5 sec at 95°C, annealing temperature for 10 sec at 58°C, extension for 5 sec at 65°C in a MiniOpticon Real-Time PCR System (Bio-Rad; USA).

The relative expression levels of each targeted gene were normalized by subtracting the corresponding beta-actin threshold cycle (C_T_) values and the fold increase (or decrease) was calculated through the 2^-ΔΔCT^ (Livak) method. Each sample was run in triplicate.

## Abbreviations

KmXR: *Kluyveromyces marxianus* Xylose Reductase; KmXDH: *Kluyveromyces marxianus* Xylitol Dehydrogenase; C5 sugar: Sugar with five carbon atoms; RNA: Ribonucleic acid; mRNA: Messenger Ribonucleic acid; cDNA: Complementary Deoxyribonucleic acid; vvm: Volume of gas per volume of batch per minute; NAD+: Nicotinamide adenine dinucleotide oxidized form; NADH: Nicotinamide adenine dinucleotide reduced form; NADP+: Nicotinamide adenine dinucleotide phosphate oxidized form; NADPH: Nicotinamide adenine dinucleotide phosphate reduced form; ROS: Reactive oxygen species; Ct: Threshold cycle; H2O2: Hydrogen peroxide; YPX: Medium containing Yeast extract, Peptone and Xylose; rpm: Revolutions per minute; PCR: Polymerase chain reaction; RTq-PCR: Quantitative reverse transcriptase polymerase chain reaction; CDW: Cell dry weight; OD: Optical density; HPLC: High pressure liquid chromatography; PI: Propidium iodide; DHR-123: Dihydrorhodamine 123; H2SO4: Sulphuric acid; N2: Nitrogen; NaOH: Sodium hydroxide; MgCl2: Magnesium chloride solution; TrisHCl: Tris hydrochloride buffer; FL1: Filter n°1; FL4: Filter n°4; PBS: Phosphate-buffered saline.

## Competing interests

The authors declare that they have no competing interests.

## Authors’ contributions

LS carried out the fermentation experiments, the flow cytometric assays, the enzymatic and the transcriptional assays, participated in the evaluation of the data and in compiling the manuscript. SP carried out the fermentation and the flow cytometric assays, participated in the evaluation of the data. LR contributed to the data interpretation and manuscript revision. DP and PB conceived the study, participated in its design, data interpretation and compiled the manuscript. All the authors have read and approved the final manuscript.
